# Percutaneous endoscopic transforaminal decompression surgery for symptomatic double-level lumbar spinal stenosis with ossification

**DOI:** 10.1097/MD.0000000000039704

**Published:** 2024-09-13

**Authors:** Jiadong Li, Xiaoping Xu, Yunjing Shui

**Affiliations:** a Department of Orthopedics Surgery, The First Affiliated Hospital of Chengdu Medical College, Chengdu, Sichuan, China.

**Keywords:** double-level, lumbar spinal stenosis, ossification, percutaneous endoscopic surgery, transforaminal decompression

## Abstract

This study aimed to explore the short-term effects of percutaneous endoscopic transforaminal decompression (PETD) for the treatment of symptomatic double-level lumbar spinal stenosis (LSS) with ossification. Twenty-eight patients diagnosed with double-level lumbar spinal stenosis who underwent double-level PETD surgery between January 2021 and January 2023 at our institution. General information, such as age, sex, disease duration, hospitalization time, and operation time, was recorded. Magnetic resonance imaging (MRI) dural sac cross-sectional area (DSCA) was recorded to assess the degree of spinal canal decompression. The White–Panjabi scoring system (WP) was used to assess preoperative and postoperative lumbar spine stability. Pre- and postoperative visual analog scale (VAS) and Oswestry Disability Index (ODI) scores were recorded to assess symptom improvement, and surgical efficacy was evaluated using the modified Macnab evaluation criteria at the 1-year postoperative follow-up. The types and risks of complications were also recorded. The patient’s 1-year postoperative follow-up MRI showed that both L3/4 and L4/5 DSCA were significantly enlarged compared with preoperative values (*P* < .001). There was no significant difference in the WP scores at 3 months postoperatively compared with those preoperatively (*P* > .05). The VAS scores for hip and lower extremity pain at 3 days, 3 months, and 1 year postoperatively were significantly lower than those preoperatively (*P* < .001), and the ODI scores at 3 months and 1 year postoperatively were significantly lower than those preoperatively (*P* < .001). There were no significant differences in hip pain, lower extremity pain VAS scores, or ODI scores between the postoperative follow-up time points (*P* > .05). There was 1 case of lower limb numbness and 1 case of neuroedematous pain in the postoperative period, and all patients had no complications, such as dural sac tear, infection, or recurrence. The 1-year postoperative follow-up was assessed as excellent in 17 cases, good in 9 cases, and possible in 2 cases using the modified Macnab criteria, with an excellent rate of 92.9%. The efficacy of double-level PETD for symptomatic double-level LSS is clear, the local stability of the lumbar spine is less affected, and the risk is low, which can reduce the chances of reoperation in patients. Thus, it is a recommended surgical procedure.

## 1. Introduction

Lumbar spinal stenosis (LSS) is the most common lumbar degenerative condition, and is mainly observed in patients aged > 60 years. The main clinical symptoms of LSS are neurogenic claudication and pain in the hips and legs, which can significantly affect the patient’s quality of life. Most patients with LSS who present with symptoms for the first time can be conservatively treated. However, surgery should be considered if its effect is poor. Simple neurodecompression (SN) and neurodecompression assisted by internal fixation and fusion (NIFF) are commonly used to treat LSS. NIFF surgery is primarily used to treat patients with severe discogenic lower back pain, lumbar instability, and high recurrence rates after PETD. However, without these factors, NIFF has no advantage over SN.^[1]^ In addition, most types of NIFF have disadvantages, such as large trauma, high cost, and long recovery time,^[2–5]^ especially for patients with double-level LSS, which will be further highlighted. SN includes traditional laminectomy and new surgical techniques such as microendoscopic discectomy (MED) and PETD, both of which have been developed recently. Muscle injury, amount of bleeding, and degree of cicatricial adhesion are more serious in traditional laminectomy than in MED and PETD,^[6]^ and complications, such as chronic low back pain and iatrogenic lumbar instability, may occur after surgery. However, the clinical effect of traditional open surgery in patients with multilevel lumbar stenosis requiring SN remains controversial.^[7]^ MED has been widely used in clinical practice, but several studies have shown that the location of the working channel of MED is limited to outside the spinal canal, which has the disadvantage of a limited operative field of vision, and it can only be operated through the posterior approach, which increases the difficulty of completely removing the ossification of the ventral side.^[8]^ As a minimally invasive spinal surgery developed in recent years, PETD can be performed under local anesthesia and has gradually become the first choice for the SN.^[9,10]^ Working sleeves of PETD can directly reach the spinal canal, intervertebral disc, and surface of nerve tissue to carry the SN more safely. Ruetten et al showed that the incidence of infection of intervertebral space, nerve root injury, dural tear, hemorrhage and scar formation of intervertebral foramen in PETD was lower than that in MED.^[9,10]^

PETD has developed rapidly since its introduction in China in the early 20th century. Studies have shown that PETD can achieve good clinical outcomes in the treatment of Lumbar Disc Herniation, including single- and double-level herniation. However, there are few reports on the use of PETD for the treatment of double-level lumbar spinal stenosis. Traditional open neurodecompression with internal fixation and interbody fusion surgery is effective for treating double-level lumbar spinal stenosis with ossification. However, traditional open surgery is not necessarily the first-choice treatment for patients without lumbar instability or discogenic lumbago. The effectiveness and indications of PETD in the treatment of double-level LSS are not clear at present because of the difficulty in confirming the responsible segment and removing the ossification. From January 2021 to January 2023, 28 cases of double-level LSS treated with PETD were collected and analyzed to explore the recent clinical efficacy, indication range, and surgical skills by combining the experimental results with our clinical experience.

## 2. Materials and methods

Clinical data of 28 patients diagnosed with double-level lumbar spinal stenosis who underwent double-level PETD at our hospital between January 2021 and January 2023 were collected and retrospectively analyzed. All the surgeries were performed by the same surgeon. General information, such as age, sex, duration of disease, hospitalization, and duration of surgery were recorded. MRI Dural Sac Cross-sectional Area (DSCA) was recorded to assess the degree of spinal canal decompression. The White–Panjabi scoring system (WP) was used to assess preoperative and postoperative lumbar spine stability. All imaging data were evaluated and measured by the same imaging physician and spinal surgeon. Preoperative and postoperative visual analog scale (VAS) and Oswestry Disability Index (ODI) scores were recorded to assess symptomatic improvement, and surgical efficacy was assessed using the modified Macnab evaluation criteria at the 1-year postoperative follow-up. The type and odds of complications were also recorded. The same surgeon recorded the return data.

### 2.1. Inclusion and exclusion criteria

Inclusion criteria: intermittent claudication with symptoms involving unilateral hip and lower extremity pain, Imaging suggestive of double-level LSS, with the responsible and operated segments being L3/4 + L4/5; meeting the indications for simple decompression surgery (no discogenic lower back pain, segmental instability, lumbar spondylolisthesis, etc), and ineffective or ineffective standardized conservative treatment for 3 months, and Decompression by double-level unilateral decompression using a surgical approach.

Exclusion criteria: revision surgery; concomitant lumbar spine infection, tumors, or fractures; and concomitant peripheral nerve disease or sacroiliac, hip, and knee arthropathy, and concomitant psychiatric or psychological disorders, and presence of contraindications to or non-acceptance of surgery; incomplete information on follow-ups or lack of regular follow-up.

### 2.2. Surgical technique

All patients were anesthetized using local anesthesia combined with intravenous dextromethomidine. The patient was placed in a prone position with the abdomen suspended, and C-arm fluoroscopy was used to locate the operative segment and mark it to determine the puncture point. Generally, the puncture points of L2–3 and L3–4 need to be 8 to 10 cm away from the line of the spinous process, 10 to 12 cm for L4–5, and 8 to 10 cm for L5/S1 because of iliac obstruction. The puncture needle is usually tilt 10–15° to the head. Specific puncture points must be accurately measured preoperatively using computed tomography and magnetic resonance imaging (MRI).

**Figure 1.**
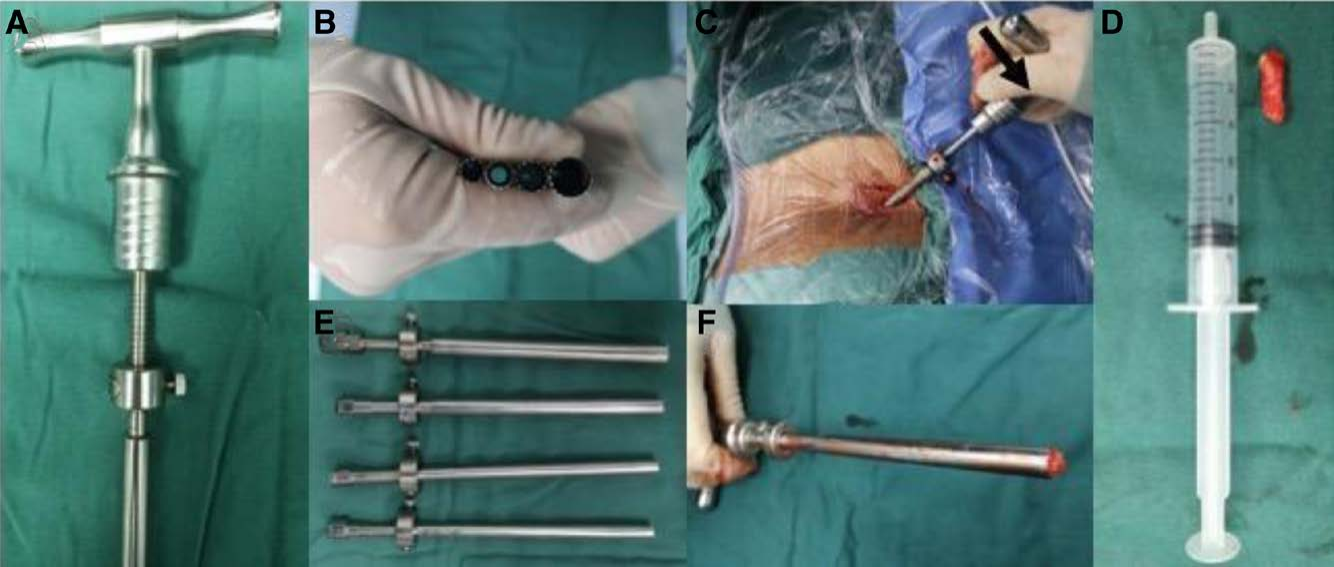
Photographs showing the limited depth trephines needed for superior arthroplasty. The trephines can achieve the purpose of depth-limiting by adjusting the knob (as shown in A), and each spiral is 2 mm. 4 types of limited depth trephines with different diameters can satisfy the needs of different forming parts (as shown in B and E). Before screwing in the limited-depth trephines, it was necessary to press down the cannulas to ensure the formation range of the back side of the SAP (as shown in C). The part of the SAP that is cut off (as shown in D). Normally the part of the SAP that is cut off can be taken out of the body following the trephines (as shown in F).

Superior facet arthroplasty was simultaneously performed at both levels. Two 18-gauge needles were inserted using a posterolateral approach after infiltration of the intended needle entry tract with 6 to 8 mL of 0.5% lidocaine. The tip of the needle was required to reach the superior articular process (SAP), and 20 mL of 0.5% lidocaine was used for infiltration anesthesia around the SAP and the intervertebral foramen. The amount of lidocaine administered can be appropriately increased according to the patient’s pain tolerance. The needles were then replaced with 2 primary guidewires with a diameter of 2 mm, and the tip of the guidewires was positioned at the tip of the SAP. After confirming that the guidewire position was satisfactory from the perspective of the C-arm, and that sequential protective cannulas were inserted over the primary guidewires, protective cannulas with a diameters of 9.5 cm. When the cannula could not enter the intervertebral foramen, its tail was tapped using a bone hammer to allow entry. The 8.5 mm-diameter limited depth trephines were used to complete the first superior arthroplasty, and the limited-depth trephine could control the entry depth with a reference interval of 2 mm according to the different forming parts (Fig. 1A). Before screwing in the limited-depth trephines, it was necessary to press down the cannulas to ensure the formation range of the back side of the SAP (Fig. 1C). Screwing the limited depth trephines gradually into the SAP from the perspective of the C arm and starting screwing in with 2 mm each time the depth reaches 10 mm and strictly limits the depth to avoid exceeding 14–16 mm, otherwise we believe that it may damage the nerves. To complete a satisfactory superior arthroplasty for a positive radiograph, the limited-depth trephines should not exceed the midpoint of the line between the spinous process and inner edge of the pedicles. For a lateral radiograph, the limited-depth trephines should not exceed the line of the posterior edge of the vertebral body. After the completion of the first superior facet arthroplasty (Fig. 1 D, F), the responsible levels were successively subjected to neurodecompression under the microscope, and the untreated responsible level was blocked with a gelatin sponge to wait for treatment.

The radiofrequency ablation electrode was used to remove the soft tissue in the operative field of vision and the bony anatomical markers, including the position of the arthroplasty, basilar part of the SAP, upper edge of the pedicle, and the yellow ligament on the dorsal side of the nerve root. Under direct vision, a 7.5 mm limited depth trephine and various other types of trephine were used to remove the basilar part of the SAP (Fig. 1B, E), and a high-speed grinding drill was used to expand the intervertebral foramen and expose the nerve root and dural sac. The bone that led to spinal canal stenosis was removed using a lamina rongeur and high-speed drill. We believe that it is more effective and safer to remove the ligamentum flavum along the dural sac and the nerve root. The lingual surface of the working cannula was used to protect the dural sac and nerve root, and a radiofrequency ablation electrode was used to shrink the flocculent soft tissue on the ventral side of the nerve and perform prehemostasis for possible bleeding points. When removing the protruding or ossified area on the ventral side, all operations must be performed under direct vision to avoid damaging the dural sac and nerve root. Decompression standard: 4 mm above the lower edge of the upper vertebral body on the head side, 6 mm above the upper edge of the lower vertebral body on the caudal side, 3 to 5 mm behind the dural sac on the dorsal side; the inner edge of the nerve root was fully exposed. After neurodecompression, the following manifestations appeared: recovery of the filling shape of the dural sac and a significant reduction in nerve root tension. The aforementioned key points of operation are then repeated at another level of responsibility.

### 2.3. Postoperative management

There was no need to place a drainage tube or to prevent postoperative infection. Endothelial sutures were used in all patients and the incision dressing was changed every 2 to 3 days. Reexamination of X-ray and computed tomography scans to determine whether the range of neurodecompression is sufficient; however, MRI is not a routine reexamination because of its unclear image due to intraoperative hemorrhage. Under the premise of wearing the waist circumference, getting out of bed, exercising properly, and under the guidance of doctors, completing the exercise of the lumbodorsal muscle and straight leg raising in the bed. On the second postoperative day, patients with obvious postoperative pain relief were discharged from the hospital on the third postoperative day; however, multimodal analgesia, dehydration, and detumescence of the nerve roots were required in some patients who experienced repeated pain due to neuroedema. All patients were required to avoid bending, twisting, weight-bearing, long-distance walking, and other activities within 1 month, and regular outpatient follow-up was completed after the operation.

### 2.4. Statistical analysis

SPSS software (version 20.0; SPSS Inc., Chicago) was used for the SPSS analysis. The measurement data line normality test was consistent with a normal distribution. Data are expressed as mean ± standard deviation, and the independent samples *t* test was used to compare non-continuous measurements before and after surgery. The count data were expressed as rates. The VAS and ODI scores at multiple time points were compared using a repeated-measures measures ANOVA. If the test of sphericity was unsatisfactory, the Greenhouse-Geisser method was used for correction, the Bonferroni method was used for comparison of different time points in the same group, and the level of the test was considered 2-sided α = 0.05.

## 3. Results

The mean age of the included patients was 55.0 ± 13.5 years, including 16 males and 12 females, with a mean disease duration of 24.9 ± 10.2 months and a mean hospitalization time of 5.1 ± 1.7 days. All patients underwent double-level unilateral decompression with PETD, the operated segments were L3/4 + L4/5, and the average operative time was 102.2 ± 14.6 minutes (Table 1). One case of lower limb numbness and 1 case of neuroedematous pain appeared after surgery and was treated with postoperative nerve nutrition, dehydration, edema reduction, and symptomatic treatment. Both patients recovered within 1 week after surgery. None of the patients had complications such as dural sac tear, infection, or recurrence, and the overall complication rate was 7.1% (Table 2).

**Table 1:** Summary of the baseline data.

Characteristics	PETD (n = 28)
Age (yr)	55.0 ± 13.5
Sex M/F	16/12
Duration of symptoms (mo)	24.9 ± 10.2
Hospital stay (d) Operating time (min)	5.1 ± 1.7 102.2 ± 14.6

n = total number of patients, PETD = percutaneous endoscopic transforaminal disctomy.

**Table 2:** Complications of PETD.

Complications	n (28)
Lower limb numbness	1
Neuroedematous pain	1
Dural sac tear	0
Infection Recurrence	0 0
Rate (%)	7.1

n = number of patients, PETD = percutaneous endoscopic transforaminal disctomy.

Preoperative MRI showed L3/4 DSCA of 132.1 ± 19.2 mm^2^ and L4/5 DSCA of 132.9 ± 16.3 mm^2^, and 1-year postoperative MRI showed that L3/4 DSCA of 237.4 ± 10.9 mm^2^ and L4/5 DSCA of 227.0 ± 12.6 mm^2^ were significantly enlarged compared with the preoperative period (*P* < .001; Table 3). The preoperative and 3-month postoperative WP scores were 2.4 ± 0.9 and 2.7 ± 1.0 points, respectively, with no statistically significant difference (*P* > .05; Table 4). The preoperative and 3-day, 3-month, and 1-year postoperative hip VAS scores were 5.5 ± 0.9, 0.9 ± 0.8, 1.0 ± 0.8, and 0.6 ± 0.6, respectively, and the lower extremity pain scores were 5.0 ± 1.4, 1.0 ± 0.9, 0.9 ± 0.9, and 0.8 ± 0.8, respectively. The VAS scores for hip and lower extremity pain were significantly lower at each postoperative follow-up time point than preoperatively (*P* < .001; Table 5), and there were no significant differences between the postoperative follow-up time points (*P* > .05). The ODI scores were 61.5 ± 5.9, 9.2 ± 2.7, and 9.3 ± 2.7, preoperatively and at three months and 1 year postoperatively, respectively. The ODI scores at each postoperative follow-up time point were significantly lower than those preoperatively (*P* < .001; Table 5), and there was no significant difference between the postoperative follow-up time points (*P* > .05). The 1-year postoperative follow-up was assessed as excellent in 17 cases, good in 9 cases, and feasible in 2 cases using the modified Macnab criteria, with an excellent rate of 92.9%.

**Table 3:** Comparison of the DSCA between pre and postoperative.

Characteristics	Pre-op	Post 1y-op	*P*
DSCA (mm^2^) L3/4 L4/5	132.1 ± 19.2 132.9 ± 16.3	237.4 ± 10.9 227.0 ± 12.6	<.001 <.001

The area of the dural sac was measured by cross-sectional MRI using the IC-PACS imaging system.

DSCA = dural sac cross-sectional area, post-op = postoperative, pre-op = preoperative.

**Table 4:** Comparison of the WP between pre and postoperative.

Characteristics	Pre-op	Post 3m-op	*P*
WP	2.4 ± 0.9	2.7 ± 1.0	.265

post-op = postoperative, pre-op = preoperative, WP = White–Panjabi Scoring System.

**Table 5:** Comparison of the VAS and ODI between pre and postoperative.

Characteristics	Pre-op	Post 3d-op	Post 3m-op	Post 1y-op	*F*	*P*
VAS Hip Leg ODI	5.5 ± 0.9 5.0 ± 1.4 61.5 ± 5.9	0.9 ± 0.8 1.0 ± 0.9 —	1.0 ± 0.8 0.9 ± 0.9 9.2 ± 2.7	0.6 ± 0.6 0.8 ± 0.8 9.3 ± 2.7	230.5 147.5 1912.8	<.001 <.001 <.001

CMM of VAS hip (pre > post 3d = post 3m = post 1y), CMM of VAS leg (pre > post 3d = post 3m = post 1y), CMM of ODI (pre > post 3m = post 1y).

CMM = comparison of multiple means, ODI = Oswestry Disability Index, post-op = postoperative, pre-op = preoperative, VAS = visual analogue scale.

### 3.1. Representative case

**Figure 2.**
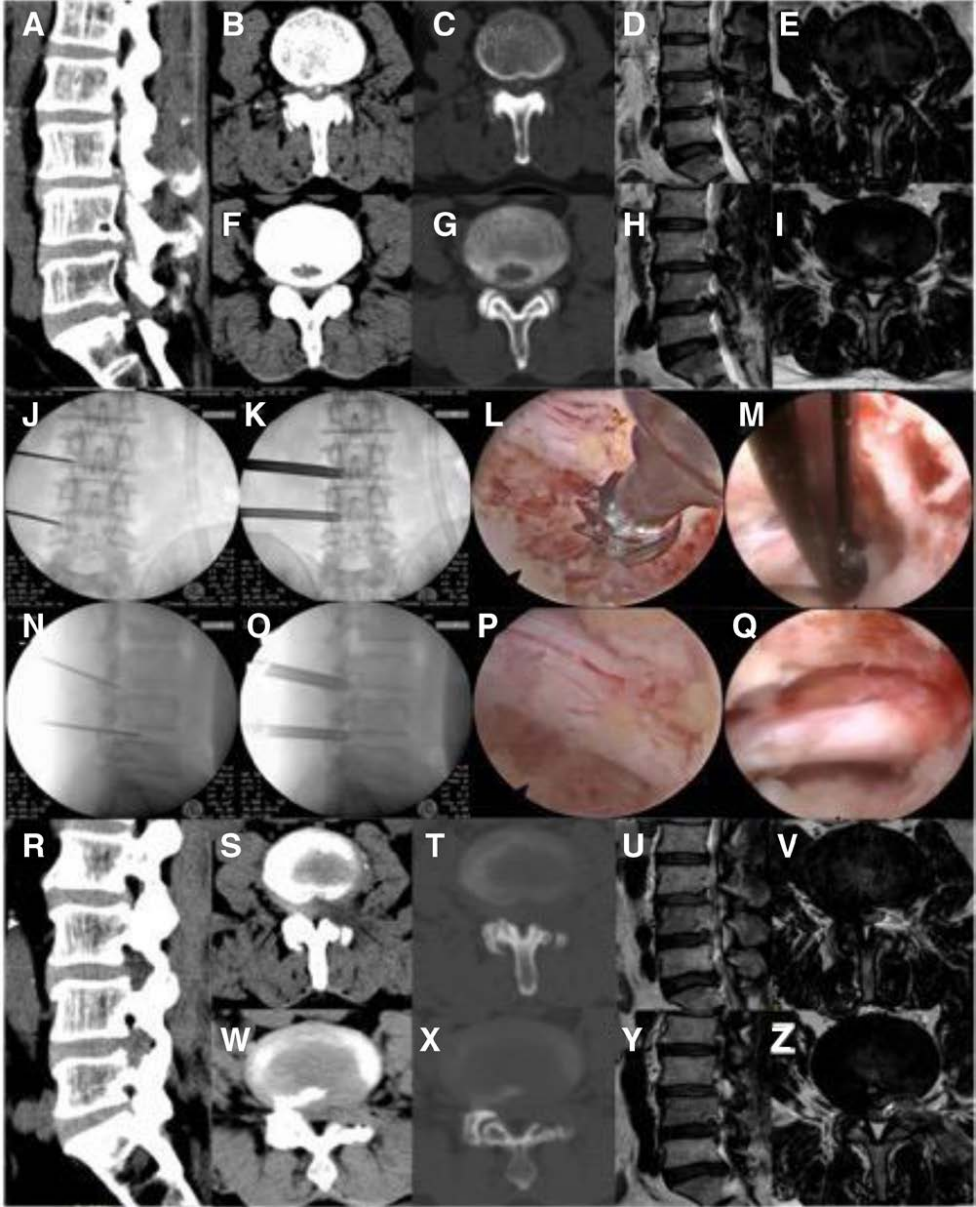
(A) patient diagnosed with double level LSS with ossification experienced double level unilateral PETD surgery. (A–I) Preoperative magnetic resonance imaging and computed tomography show intervertebral disc calcification in L3/4 and posterior edge separation in L4/5. (R–Z) Postoperative magnetic resonance imaging and computed tomography show that double level ventral part of the SAP, prominent nucleus pulposus and ventral ossification of dural sac and nerve root have been removed. The lateral recess and neuroforamen are enlarged. (J–Q) Superior arthroplasty was performed simultaneously on both levels. Endoscopy showed that the nerve roots were fully decompressed in L3/4 (L, M) and L4/5 (P, Q).

A representative case is shown in Figure 2.

## 4. Discussion

### 4.1. Effectiveness of double-level PETD in the treatment of double-level LSS

Degenerative lumbar spinal stenosis (DLSS) is one of the main causes of low back pain and lower limb dysfunction, and some patients experience a serious impact on their quality of life. Surgical decompression is the most effective treatment option in patients with poor outcomes. For patients without discogenic lower back pain, lumbar instability, or lumbar spondylolisthesis, simple nerve decompression is the first-choice surgery. In recent years, single large-scale simple decompression has been gradually abandoned and replaced by minimally invasive limited decompression after defining the responsible segment and responsible nerve root, which can avoid excessive intraoperative resection of bony structures, maintain spinal stability, and improve the long-term efficacy of surgery. Single-segment PETD is widely used for DLSS, with minimal surgical trauma, rapid recovery, and satisfactory therapeutic results. There is no clear conclusion regarding the treatment of multisegmental DLSS, and some researchers believe that multiple surgeries should be used to decompress patients individually until their efficacy is satisfactory.^[11]^ However, an increase in the medical costs and surgical risks associated with multiple surgeries remains unavoidable.

Some patients with multisegmental DLSS may have multiple nerve compressions; therefore, decompression of only one nerve may result in poor symptom relief. In this trial, some patients’ pain involved multiple nerve root alignment areas, and we chose dual-segment PETD to thoroughly explore and decompress all involved nerve roots. All patients in this trial experienced a significant reduction in their postoperative VAS and ODI scores, which also demonstrated that dual-segment PETD could effectively ensure the clinical efficacy of the postoperative treatment. In addition, it should be noted that the nerve root may be compressed for different pathological reasons during its entire journey from the dural sac to the intervertebral foraminal area.^[12]^ We believe that, for some patients with only a single responsible nerve root, we may clarify whether there is compression in parts of the body other than the responsible segment. MRI is the preferred imaging test for diagnosing DLSS, and combined with selective neurographic block, can effectively suggest the site of nerve root compression, including the lateral saphenous area, central canal area, and intervertebral foraminal area. Some researchers agreed that selective neurographic block can quickly and effectively clarify the responsible nerve root.^[13,14]^ However, not all compressions present with clinical symptoms, and Eastley concluded that selective nerve root block has a sensitivity of 93% but a specificity of only 26%.^[15]^ Therefore, to ensure clinical efficacy, limited expansion of the decompression range and segments can be an effective measure for improving the efficacy of double-level DLSS surgery. In this trial, 4 patients were clearly identified as lumbar 4 as the responsible nerve root by selective nerve root angiography block, but imaging examination suggested that compression existed in the L3/4 lateral saphenous fossa region and L4/5 intervertebral foraminal area, and all of them were satisfied with the efficacy of double-level PETD.

### 4.2. Safety of double-level PETD for the treatment of double-level LSS

PETD uses a transforaminal approach to maximize protection of the nerves in the spinal canal and normal spinal structures and to reduce interference with the neural tissues; therefore, the procedure can usually be performed under local anesthesia. Surgery under local anesthesia also allows the patient to provide feedback to the operator, effectively reducing the risk of nerve root injury and dural sac tear. None of the patients in our group had a reduction in muscle strength after surgery, but there was 1 case of neuroedematous pain and 1 case of numbness in the lower limbs, which we believe was related to intraoperative stimulation of the nerves. The 2 patients also provided clear feedback during the operation, which did not result in irreversible nerve damage. In addition, local anesthesia surgery resulted in a shorter hospital stay and a lower cost of surgery than general anesthesia surgery. When Mayer performed lumbar endoscopic surgery in 30 patients with lumbar disc herniation, he found that surgery under local anesthesia could reduce hospitalization time, postoperative complications, and other advantages.^[16]^ Asano et al^[17]^ also treated lumbar disc herniation using spinal endoscopic surgery under local anesthesia and concluded that surgery under local anesthesia has the advantages of shorter hospitalization time and faster recovery.

Steps in PETD that require fluoroscopic assistance include puncture anesthesia, channel placement, and foraminoplasty. An increase in the number of segments operated on may lead to an increase in the number of fluoroscopies and radiation doses, which may cause greater harm to the operator, operating room personnel, and patients.^[18]^ Patients with 2-segment DLSS who underwent single-segment PETD were exposed to additional fluoroscopy and radiation when a second PETD procedure was required in the event of a poor postoperative outcome. In this trial, we performed intraoperative foraminoplasty on both segments simultaneously based on the clarification of the responsible segments, which can effectively reduce the number and time of fluoroscopy and lower the radiation dose. Some studies have suggested that C-arm fluoroscopy radiation can have negative psychological effects on the operating room staff, including developing diseases and affecting fertility.

Symptoms may reappear in some patients after PETD, and pathological changes may manifest as lesions in the same or other segments, which we refer to as recurrence in this study.^[19]^ We believe that adequate preoperative analysis of the different compression sites of the responsible nerve roots and accurate determination of the different responsible nerve roots are key steps in avoiding postoperative recurrences. Double-level PETD provides more adequate and comprehensive decompression of potentially compressed nerve roots through preoperative imaging and nerve root block, which can reduce the chance of short-term postoperative recurrence in patients owing to the insufficient treatment scope. In this trial, none of the patients experienced recurrence during the 1-year follow-up period, which also proves the value of dual-segment PETD in preventing postoperative recurrence.

## 5. Conclusion

The efficacy of double-level PETD for symptomatic double-level LSS is clear, the local stability of the lumbar spine is less affected, and the risk is low, which can reduce the chances of reoperation. Therefore, it is recommended as the surgical procedure. The sample size of this study was relatively small, and we hope that a systematic prospective pilot study will be conducted at a later stage to clarify the clinical application of double-level PETD further.

## Author contributions

**Data curation:** Jiadong Li, Yunjing Shui.

**Methodology:** Xiaoping Xu.

**Writing – original draft:** Jiadong Li, Yunjing Shui.

**Writing – review & editing:** Jiadong Li.
